# The Prognostic Effect of Dexamethasone on Patients With Glioblastoma: A Systematic Review and Meta-Analysis

**DOI:** 10.3389/fphar.2021.727707

**Published:** 2021-08-31

**Authors:** Lingling Zhou, Yang Shen, Tingting Huang, Yangyang Sun, Raphael N. Alolga, Gang Zhang, Yuqiu Ge

**Affiliations:** ^1^ Department of Orthopaedic Surgery, Children’s Hospital of Nanjing Medical University, Nanjing, China; ^2^ Department of Urology, The Second Affiliated Hospital of Nanjing University of Chinese Medicine, Nanjing, China; ^3^ Department of Neurology, Children’s Hospital of Nanjing Medical University, Nanjing, China; ^4^ Clinical Metabolomics Center, China Pharmaceutical University, Nanjing, China; ^5^ Department of Public Health and Preventive Medicine, Wuxi School of Medicine, Jiangnan University, Wuxi, China

**Keywords:** dexamethasone, glioblastoma, prognosis, epidemiology, meta-analysis

## Abstract

**Background:** Dexamethasone (DEX) is widely adopted to reduce tumor-associated edema in glioblastoma (GBM) patients despite its side effects. However, the benefits of using DEX in GBM patients remains elusive.

**Methods:** In this study, we performed a comprehensive meta-analysis to address this concern. We searched the relevant studies from PubMed, Web of Science, and EMBASE databases, and then applied random or fixed-effects models to generate estimated summary hazard radios (HRs) and the 95% confidence intervals (CIs). Moreover, subgroup and sensitivity analysis were conducted and publication bias were further evaluated.

**Results:** Ten articles with a total of 2,230 GBM patients were eligible according to the inclusion criteria. In the assessment of overall survival (OS), meta-analysis data revealed that DEX was significantly associated with the poor prognosis of GBM patients (HR=1.44, 95% CI=1.32−1.57). In the progression-free survival (PFS), the pooled results indicated that the use of DEX can increase 48% death risk for GBM patients (HR=1.48, 95% CI=1.11−1.98). Subgroup analyses revealed that DEX was associated with poorer outcome of GBM in subgroup of newly diagnosed patients and GBM patients treated with ≥ 2mg/day. Sensitivity analyses showed that no study changed the pooled results materially for both OS and PFS analyses. The funnel plot had no obvious asymmetry.

**Conclusion:** Our findings partly confirmed that use of DEX was associated with poor treatment outcome in GBM patients. To reach a definitive conclusion, large samples from multi-centers are urgent to address this concern.

## Introduction

Glioblastoma (GBM) is the most common form of primary brain tumors among adults (Wen and Kesari, 2008), with an extremely poor median survival outcome of 15.3–21.7 months (Hegi et al., 2005). Symptomatic peritumoral edema is frequently caused by GBM, whereby, blood-brain barrier dysfunction allows fluid into the extracellular space of the brain parenchyma. Dexamethasone (DEX) is a synthetic corticosteroid with a wide range of biological functions, including powerful anti-inflammatory effects (Arvold et al., 2018). Many studies have reported that the use of DEX effectively improves neurologic symptoms caused by GBM (Vecht et al., 1994; Dubinski et al., 2019).

Currently, a few clinical studies have been conducted to investigate the use of DEX in the treatment of GBM (Galicich and French, 1961; Galicich et al., 1961; Iorgulescu et al., 2021; Nayak et al., 2021). DEX has become a good choice in neurotumor treatment owing to its strong effects against tumor-induced encephaledema. DEX is reported to play the dual role of tumor-suppression and promotion. Other studies have associated the use of DEX with systemic toxicities such as, hyperglycaemia, gastrointestinal perforation and bleeding with or without infection (Heimdal et al., 1992; Dubinski et al., 2019). Although it has not been shown that DEX directly interferes with the therapeutic efficacy of anti-glioblastoma, mounting evidence from clinical and laboratory data suggest that it may affect the patient’s anti-tumor immunity (Wong et al., 2015). According to Reardon’s group, DEX therapy is linked to the poor outcome of GBM patients, its use may be detrimental to immunotherapeutic approaches (Iorgulescu et al., 2021). But Lee *et al.* reported that the use of DEX did not influence the overall survival (OS) of newly diagnosed GBM patients (Lee et al., 2020). Furthermore, a study by Dubinski *et al.* revealed that administration of DEX did not remarkably affect OS and progression-free survival (PFS) in GBM patients when compared to the controls (Dubinski et al., 2018).

DEX therapy has been used in GBM patients for decades but its prognostic effect remains controversial. In this study, we applied a meta-analysis to quantify the relationship between the use of DEX and outcome of GBM patients by summarizing the results of published cohort studies. Our findings may facilitate decision making and guide clinicians in the treatment for GBM patients.

## Materials and Methods

### Literature Search Strategy

The relevant literatures up to January 20, 2021, were retrieved from PubMed, Web of Science and embase databases. We used the following keywords: (dexamethasone OR DEX) AND (glioblastoma OR GBM) to obtain the relevant studies. This systematic review was accomplished according to the Preferred Reporting Items for Systematic Reviews and Meta-Analyses (PRISMA) guidelines, as shown in Supplementary File S1.

### Study Inclusion Criteria and Selection

To reduce the differences between the retrieved studies, we applied the following selection criteria: 1) Studies that provided the necessary information to calculate the hazard ratio (HR) related to GBM survival; 2) To avoid selection bias, the sample size of every study had to be more than 20; 3) The median follow-up time were more than 12 months. If there were two or more studies published in the same population, the most informative or latest article was chosen. Two researchers (Lingling Zhou and Yang Shen) evaluated the relevant studies according to the predetermined criteria independently. The discrepancies were resolved through discussion.

### Data Extraction and Quality Assessment

In each study, the following information was acquired: First author, PMID, publication year, country, the median of follow-up time, study type, median age, male percentage, No. of MGMT methylation (DEX user/no user), baseline information, DEX dose, clinical and neurological data, location and extent of the lesion, the extent of surgical resection and the volume of tumor residue, the use of adjuvant treatments, and IDH mutation, HR adjustment variables and the corresponding 95% confidence interval (CI). Data were extracted independently by two authors (Yangyang Sun and Yang Shen) and then cross-checked.

The Newcastle-Ottawa Scale evaluation system was employed to score the quality of eligible studies (Stang, 2010). The scale had a maximum score of ten to assess all the studies based on the selection of participations, the comparability of populations and the measurement of exposure or the ascertainment of outcomes of interest. A study with a score ≥7 was treated as high-quality.

### Statistical Analysis

We used a random or fixed-effect model to assess the relationship between the use of DEX and the survival of GBM patients. The heterogeneity among studies was estimated by *Q*-test and *I* ^2^ value. Random-effect model was used if observed *Q*-test *p* < 0.10 or *I* ^2^ > 50%. Otherwise, the fixed-effect model was applied. We conducted subgroup analyses to assess possible confounding factors of these variables. A pooled HR was calculated to assess the survival effect of DEX on GBM. By deleting every study in turn, the sensitivity analysis was performed to evaluate the stability of this systematic study. The Egger’s test and Begg’s funnel plot was used to determine the publication bias. A two-sided *p* value less than 0.05 was regarded as statistically significant. The analyses were conducted using R software (R-3.5.1) and the package name is ‘metafor’.

If the survival information was reported by Kaplan-Meier curve and did not provide HR value and corresponding 95% CI, we used GetData Graph Digitizer and Engauge digitizer software to get survival data. Besides, we calculated the HR and 95% CI according to the observed events of the group that used DEX and those who did not and the *p* value for the log-rank test when the studies did not provide the HR.

## Results

### Search Results, Study Selection and Quality Assessment

**FIGURE 1:**
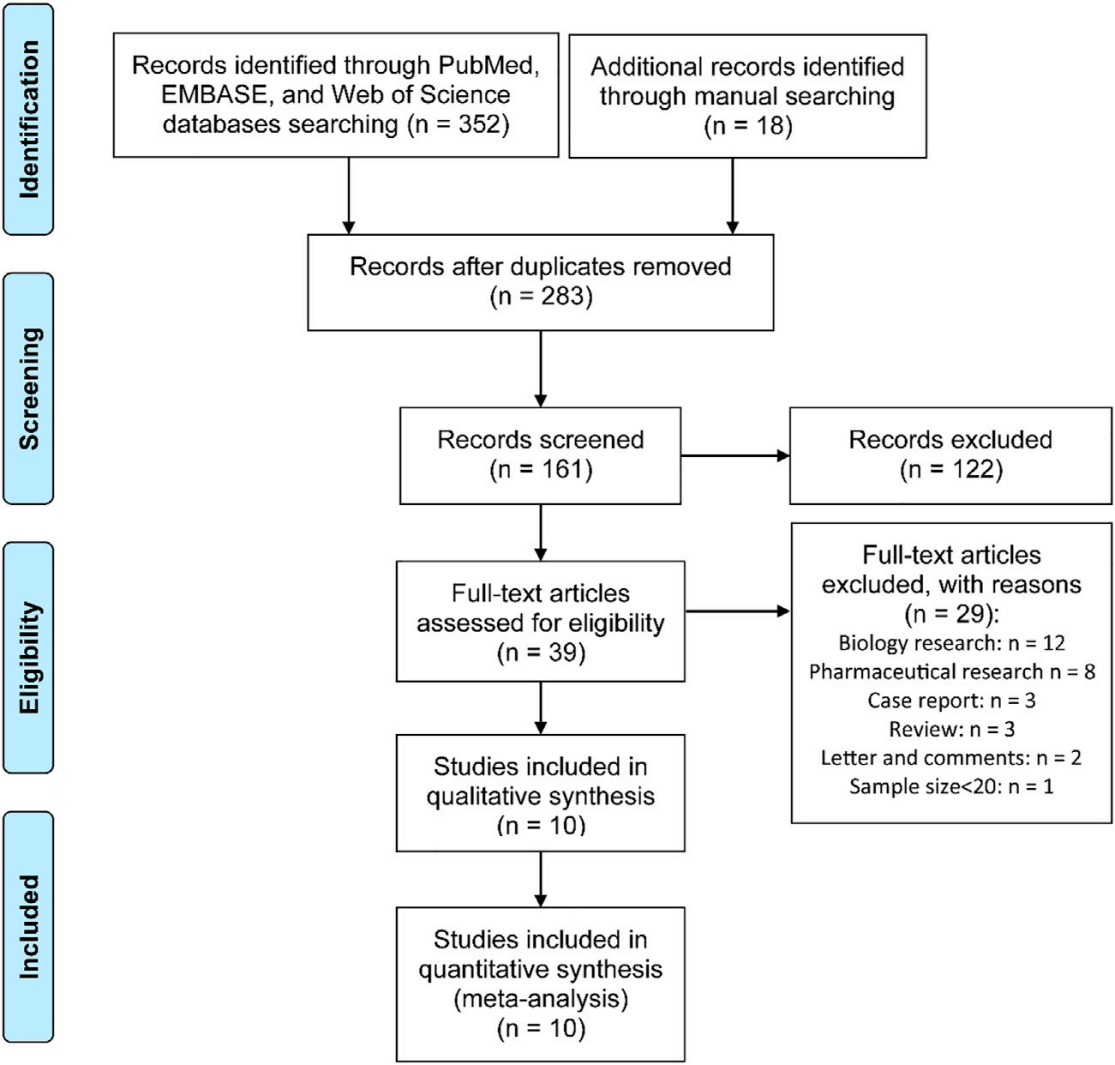
PRISMA flow diagram for the process of study selection.

A total of 370 relevant articles were retrieved from the database search. Among them, 283 were eligible for further screening. After abstract review, 122 articles were excluded. In the remaining 161 literatures, 122 incompetent studies were excluded after full-text assessment. From the remaining 39 articles, 29 were further excluded based on the following reasons: biology research (n = 12), pharmaceutical research (n = 8), case report (n = 3), review (n = 3), letter and comments (n = 2). The work of Vivien Tang *et al.* was excluded due to the small sample size (<20) to eliminate potential selection bias (Tang et al., 2008). Finally, 10 articles (Derr et al., 2009; Shields et al., 2015; Wong et al., 2015; Bhavsar et al., 2016; Dubinski et al., 2018; Hui et al., 2019; Lewitzki et al., 2019; Lee et al., 2020; Iorgulescu et al., 2021; Nayak et al., 2021) were eligible for this meta-analysis (Figure 1).

The 10 studies were published between 2009 and 2020 and had involved 2,230 GBM patients from two prospective cohort studies and eight retrospective cohort studies in three countries. The selected articles had a sample size that ranged from 35 to 841. The median follow-up time was diverse from 14.6 to 49.4 months. Six cohorts detected the status of MGMT methylation. Among the patients with MGMT methylation, 188 were in the group that used DEX whereas 228 were not (Table 1).

**TABLE 1:** The main characteristics of included studies.

First author	Publication year	Country	Median of follow-up (months)	Median age	Male/All patients	MGMT methylation (user/no user)	Newly diagnosed	Clinical information	Adjustment variables
Iorgulescu *et al.*	2020	United States	22.1	57.5 (50.3, 64.6)	102/181	37/22	No	DEX dose: (1 or 2.5 mg/kg) or high (10 mg/kg)/day. Patients: 181 GBM with IDH wild-type and treated with PD-(L)1 blockade; 75.7% at recurrence and 24.3% in the newly diagnosed setting	Disease setting, age, tumor size, tumor resection, MGMT methylation and tumor volume, and extent of resection
Nayak *et al.*	2020	United States	48.6, 49.4	53 (42, 60)	54/80	20/9	No	DEX dose: 4 mg/day. Patients: 80 GBM treated with PD-(L)1 blockade; 13 patients with resection prior to study; 88.8% Grade IV glioma; 70% IDH1 status	None
Lee *et al.*	2020	Korea	26.1	59.0 (20, 79)	67/125	15/35	Yes	DEX dose: 2–4 mg/day. Patients: 186 newly diagnosed GBM treated with surgical resection; 4% with IDH1 mutation, 28.8% with radiation therapy	Sex, lymphopenia, incomplete resection, non-GTR and an ECOG score
Lewitzki *et al.*	2019	Germany	20.3	59 (11, 81)	92/152	12/35	Yes	DEX dose: no report. Patients: 229 patients with GBM treated with Radio-chemotherapy; 32.9% treated with complete resection; 3.9% with IDH-mutation	RT-protocol NFRT, salvage, MGMT methylation, secondary GBM
Hui *et al.*	2019	United States	14.6	57 (21, 82)	194/319	83/83	Yes	DEX dose: 2 and 4 mg/day. Patients: 319 newly diagnosed and nonmetastatic GBM who received standard photon RT; the majority of patients received concurrent TMZ at a dose of 75 mg/m^2^	Age, extent of resection, and MGMT methylation
Dubinski *et al.*	2018	Germany	15.5	58 (12.72)	59/113	21/44	Yes	DEX dose: 12 mg/day. Patients: 113 newly diagnosed GBM; 38% located in temporal and 34% in parietal; 15% with IDH mutation; 54% with GTR resection	None
Bhavsar *et al.*	2016	United States	18.1	56.07 (12.63)	674/841	None	Yes	DEX dose: no report. Patients: 841 GBM underwent primary brain tumor resection; 96.1% with radiation	Age, gender, BMI, ASA physical status, and CCI score
Shields *et al.*	2015	United States	15.6	61 (28, 79)	44/73	None	Yes	DEX dose: no report. Patients: 73 patients with GBM; 34 with adjuvant BEV and 39 with TMZ or TMZ alone; 51% with dexamethasone during RT.	BEV, extent of resection, age, gender, XRT dosage, smoking status, and BMI
Wong *et al.*	2015	United States	39	57 (30, 77); 54 (24, 80)	22/35; 92/120	None	No	DEX dose: > 4.1 mg per day. Patients: 155 recurrent GBM; 91% patients >2 times recurrence; 58 patients treated with PD-(L)1 blockade	None
Derr *et al.*	2009	United States	No reports	55.7 (11.2)	95/191	None	Yes	DEX dose:10 mg/day. Patients: newly diagnosed GBM.	Mean glucose, age, and KPS

OS, over survival; PFS, progression-free survival. DEX, dexamethasone; GBM, glioblastoma.

The scores of quality assessment were shown in Table 2. All articles were of high quality with a quality score of 7–9.

**TABLE 2:** Methodologic quality of cohort studies included in the meta-analysis.

Study (PMID)	Representativeness of the exposed cohort	Selection of the nonexposed cohort	Ascertainment of exposure	Outcome of interest not present at start of study	Control for important factors	Assessment of outcome	Follow-up period long enough for outcomes to occur^a^	Adequacy of follow-up evaluation of cohorts^b^	Total quality scores
33239433	★	★	★	★	★	★		★	7
33199490	★	★	★	★		★	★	★	7
32648384	★	★	★	★	★	★	★★	★	9
31831026	★	★	★	★		★	★★	★	8
30864102	★	★	★	★	★	★	★	★	8
29349612	★	★	★	★		★	★★	★	8
27396375	★	★	★	★	★	★	★★	★	9
26520780	★	★	★	★	★	★	★	★	8
26125449	★	★	★	★		★	★	★	7
19139429	★	★	★	★	★	★		★	7

a A cohort study with a follow-up time longer than 5 years was assigned additional one star.

b A cohort study with a follow-up rate greater than 80% was assigned additional one star.

### Use of DEX and GBM Survival

**FIGURE 2:**
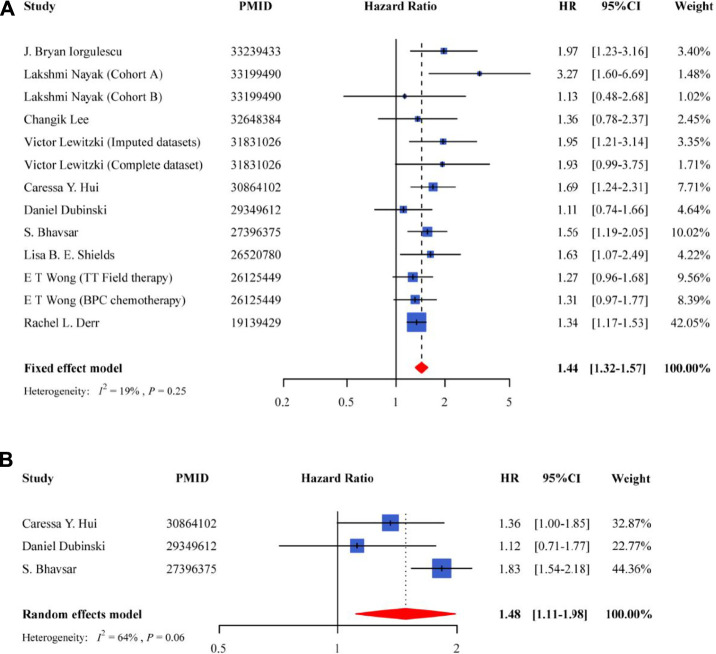
Forest plot for the association between the use of DEX and overall survival of GBM patients **(A)** and progression-free survival of GBM patients **(B)**.

For the OS, meta-analysis of the 10 studies showed that the use of DEX was associated with poor prognosis of GBM patients in the fixed-effect model (HR = 1.44, 95% CI = 1.32–1.57, *p* = 3.44E-16) (Figure 2A), and had a low heterogeneity (*I* ^2^ = 19%, *P*_heterogeneity_ = 0.25). Patients treated with DEX increased death risk by 44%. Among the studies, three provided the information on PFS, and their pooled results indicated that PFS was significantly lower in GBM patients using DEX compared to the non-users (HR = 1.48, 95% CI = 1.11–1.98, *p* = 7.31E-03) (Figure 2B).

**FIGURE 3:**
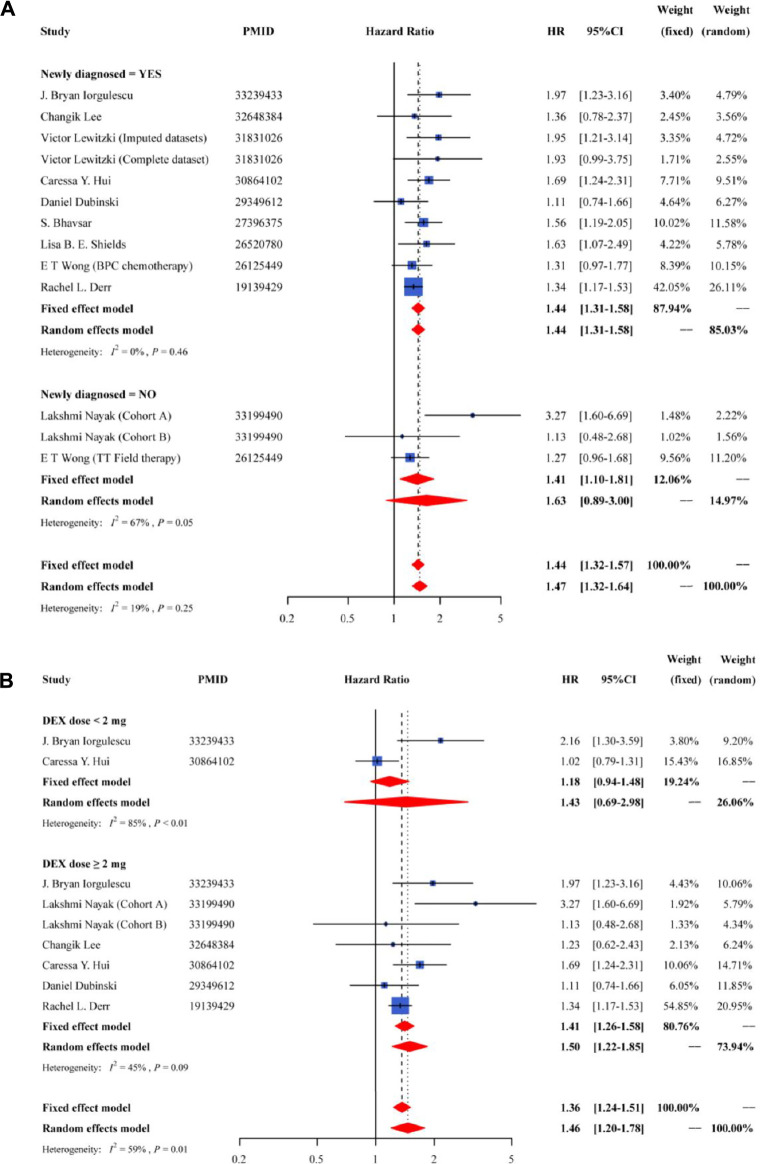
Forest plot of the pooled HR and 95% CI for OS in subgroup of newly diagnosed or not **(A)** and subgroup of different DEX dose **(B)**.

We applied subgroup analyses to evaluate the sources of potential heterogeneity or whether relationships were limited to specific population (Figure 3). The subgroup analyses based on the disease status (new or recurrent) had a significant association in the newly diagnosed group (HR = 1.44; 95% CI = 1.31–1.58), with a low heterogeneity (Figure 3A). When stratified according to DEX dose, the HRs were 1.43 (95% CI = 0.69–2.98) for <2 mg/day group and 1.50 (95% CI = 1.22–1.85) for ≥2 mg/day group, with a moderate heterogeneity (Figure 3B).

Nearly all the studies focused on specific subpopulations of glioblastoma, many with recurrent disease and on trials for immune checkpoint inhibitors. To explore the interaction between immune checkpoint inhibitors and DEX, we performed additional analysis. When stratified by whether or not underwent treatment with immune checkpoint inhibitors, the results indicated that whether treated or not with anti-PD-1 therapy, the worse effect of DEX on GBM prognosis remained be significant (HR = 1.55, 95%CI: 1.16–2.06, for PD-1 treatment; HR = 1.43, 95%CI: 1.30–1.58, for not PD-1 treatment) (Supplementary File S2).

### Sensitivity Analyses and Publication Bias

**FIGURE 4:**
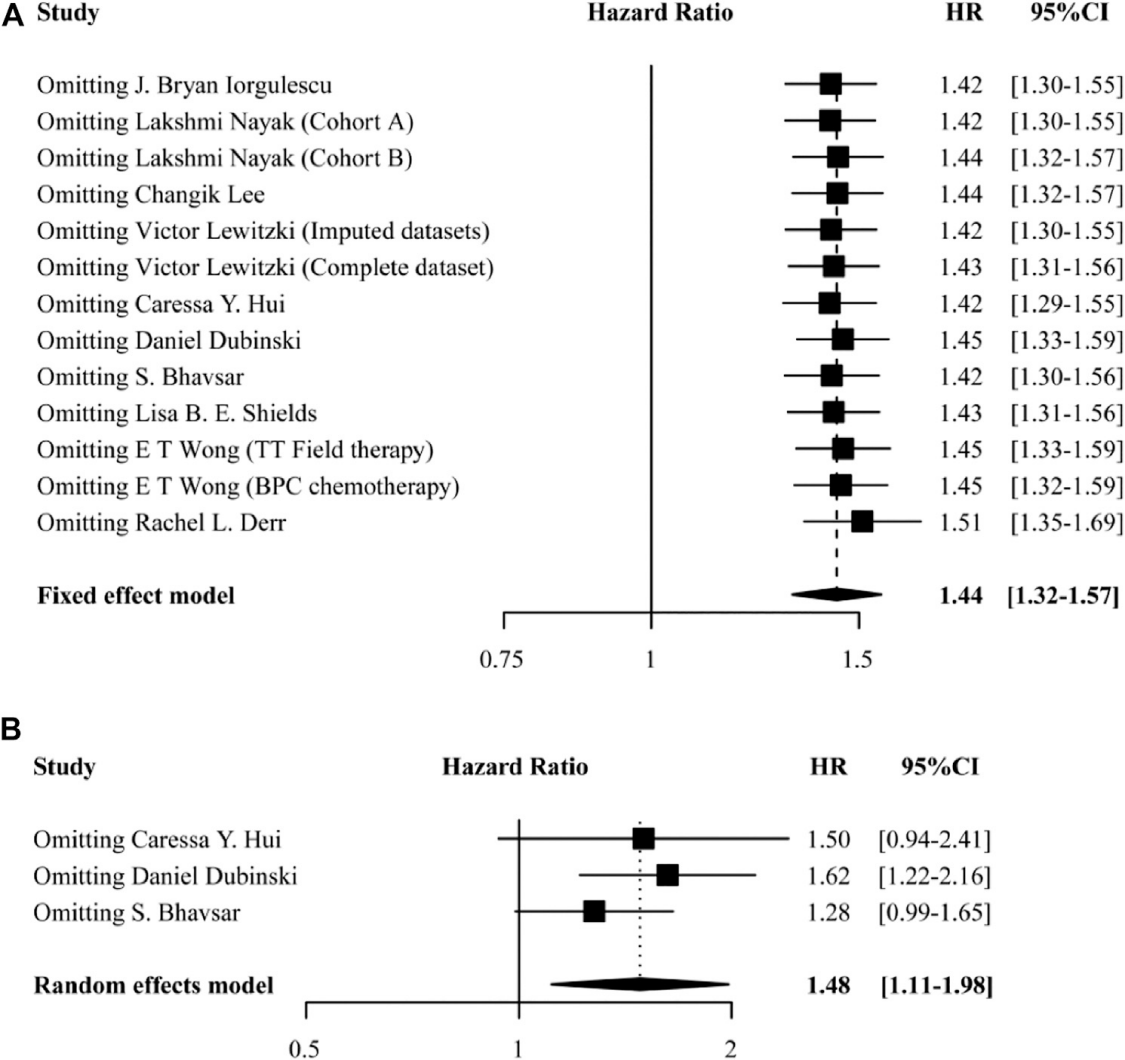
Sensitivity analysis showing the reliability of the pooled result for OS **(A)** and PFS **(B)**.

**FIGURE 5:**
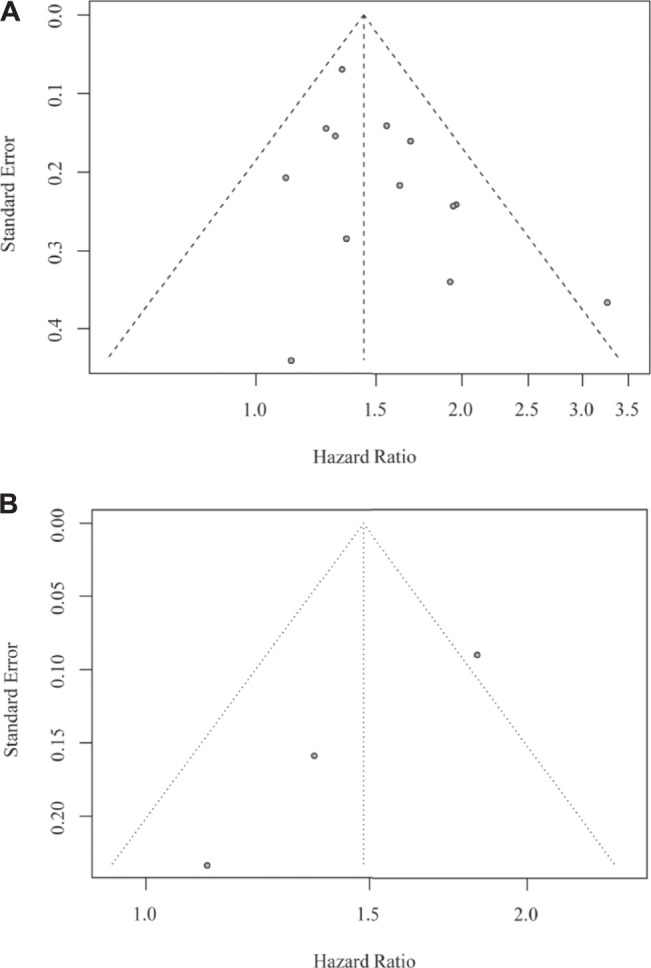
Funnel plot for the study on OS **(A)** and on PFS **(B)** of GBM patients.

We conducted sensitivity analyses to examine the essential association between the use of DEX and GBM outcome by removing each study in turn. None of the studies changed the pooled results materially in both OS (Figure 4A) and PFS analyses (Figure 4B). In addition, no remarkable asymmetry was observed in the funnel plot (Figures 5A,B). The results of Egger’s test demonstrated that there was no significant publication bias (both *p* > 0.05). These results indicated that this meta-analysis was stable and reliable.

## Discussion

To the best of our knowledge, this is the first meta-analysis to explore the relationship of DEX use and GBM prognosis. In this large and multi-centers meta-analysis of 10 observational longitudinal studies (involving 2,230 GBM patients from different countries), we found that the presence of DEX conferred an HR of ∼1.44 for those taking DEX compared to non-users. This positive association was also found in the PFS analysis. The findings demonstrated that the use of DEX may worsen the disease conditions and increase the risk of death for GBM patients. The treatment of encephaledema using DEX should be avoided. Thus, the development of new therapeutic drugs for edema is urgently needed.

The possible reasons for using DEX in clinical treatment were as follows. Previous studies have shown that the use of DEX in the clinic can cause ultrastructural alterations of the cerebral vasculature. Hedley-Whyte *et al.* found that GBM patients tissues exhibit extracellular space expansion and astrocyte vascular foot process swelling in the adjacent brain tissue, but these alterations were absent after treatment with DEX (Hedley-Whyte and Hsu, 1986). Another study found T1 relaxation of magnetic resonance imaging showed an attenuation of 13, 33, and 57% in 1, 3, and 7 days respectively, after treatment with DEX (Andersen et al., 1993). These radiologic and histologic data together demonstrated the view that effective and rapid effects of DEX in the reduction of cerebral edema caused by cerebral tumors can bring clinical benefits, therefore revealed its main indication for use in GBM patients.

Several studies have investigated the potential mechanism of DEX in the treatment of cerebral edema. It has been reported that DEX ameliorated cerebral edema by reducing the permeability of blood-brain barrier, through regulating the expression level of occludin, claudin and vascular endothelial (VE)-cadherin (Cenciarini et al., 2019). Besides, previous study revealed that DEX modulated vascular endothelial growth factor (VEGF) and angiopoietin-1, which play important roles in regulating the stabilization of blood-brain barrier (Kim et al., 2008). Other studies found that DEX altered the expression of K^+^ channel or potassium channel, leading to relieve cerebral edema (Gu et al., 2007; Gu et al., 2009).

However, studies have shown that up to 50% of patients exhibited adverse effects when treatment with DEX for peritumoral edema in GBM and brain metastasis. The top three systematic adverse effects are cushingoid appearance, hyperglycemia and psychiatric symptoms (Hempen et al., 2002; Derr et al., 2009; Arvold et al., 2018). Through attenuating the innate and adaptive immune systems, prolonged exposure to DEX could inhibit immune effector response against the GBM. According to Dubinski *et al.*, DEX could induce leukocytosis and affect the tumor immune infiltration and the survival of GBM patients (Dubinski et al., 2018). A study by Iorgulescu *et al.* indicated that DEX therapy may be detrimental to anti-PD-1 therapy for GBM patients (Iorgulescu et al., 2021). Another study by Swanson’s group found that DEX had a far-reaching impact on the efficacy of TTFields and chemotherapy, thereby reducing the OS time of patients (Wong et al., 2015).

In the present study, we conducted subgroup analysis and subgroups were based on the disease status (new or recurrent), different DEX dose (<2 mg/day group or ≥2 mg/day group), treated with anti-PD-1 therapy (yes or no). A significant association was observed in the newly diagnosed group. In addition, the results indicated that whether treated or not with anti-PD-1 therapy, DEX treatment was significantly related to worse prognosis of GBM. There was also a significant association between the use of DEX and poorer GBM survival in the DEX ≥ 2 mg/day group, but not observed in the DEX < 2 mg/day group. A previous study demonstrated that GBM patients with higher DEX doses had remarkably shorter OS than those with lower DEX doses (Wong et al., 2015). In the present study, only two studies in the DEX < 2 mg/day group were included in the subgroup analysis. The number of studies were relatively small, which may influence the result. Future studies of the prognostic effect of DEX for GBM parients treated with < 2 mg/day may be warranted. Besides, it is noteworthy that the median of follow-up in the study of Nayak *et al.* was longer than other studies, which may influence the OS of GBM patients. However, the pooled results showed significant association between the use of DEX and GBM survival after excluding the study of Nayak *et al.*

This study has some noteworthy strengths. Being a meta-analysis, it can provide a more comprehensive evidence than a separate study. The total number of subjects is very large, which greatly improved the statistical power of this systematic study. The analyses of sensitivity and publication biases showed no study that could change the results of this meta-analysis, making it stable and reliable. The low heterogeneity indicated that the synthesis of the different studies was reasonable.

This meta-analysis has some limitations. First, due to the observational studies, we cannot rule out the possibility that other potential factors may lead to the observed associations. Second, the DEX dose range varied greatly between studies, which may result in the heterogeneity of pooled analysis. Third, some studies did not provide HRs value and 95% CIs, but only showed the results of log-rank tests or Kaplan-Meier curves. Therefore, the data was digitized and extracted by a software, which could result in some imprecisions and inaccuracies. Fourth, the dose-response relationship between the use of DEX and the outcome of GBM have not been assessed due paucity of relevant studies. Fifth, in this meta-analysis, we gathered a very heterogeneous group of reports, consisting of some different types of methodology and patient selection criteria. It may result in bias of results. In this regard, more observational studies using standardized DEX strategies are urgent.

## Conclusion

In summary, the quality of observational evidence is high. The results suggest that the use of DEX was associated with shorter OS and PFS of GBM patients, especial for newly diagnosed patients or GBM patients treated with DEX ≥ 2 mg/day. Therefore, the treatment of GBM should consider a restrictive dosage of DEX or use other drugs to reduce edema based on the condition of patients.

## Data Availability

The original contributions presented in the study are included in the article/Supplementary Material, further inquiries can be directed to the corresponding authors.

## Abbreviations

DEX, dexamethasone; GBM, glioblastoma; HR, hazard radios; CIs, confidence intervals; OS, overall survival; PFS, progression-free survival; PRISMA, Preferred Reporting Items for Systematic Reviews and Meta-Analyses.
