# Supplementary material for: The Prognostic Effect of Dexamethasone on Patients With Glioblastoma: A Systematic Review and Meta-Analysis
Source: Front Pharmacol. 2021 Aug 31;12:727707. doi: 10.3389/fphar.2021.727707 (PMC8438116; doi:10.3389/fphar.2021.727707)
Supplement: Supplementary file 1 [file DataSheet2.PDF]

Supplementary Figure legends

Fig. S1. Forest plot of the pooled HR and 95% CI for OS in subgroup of PD-1 or not.

Fig. S1

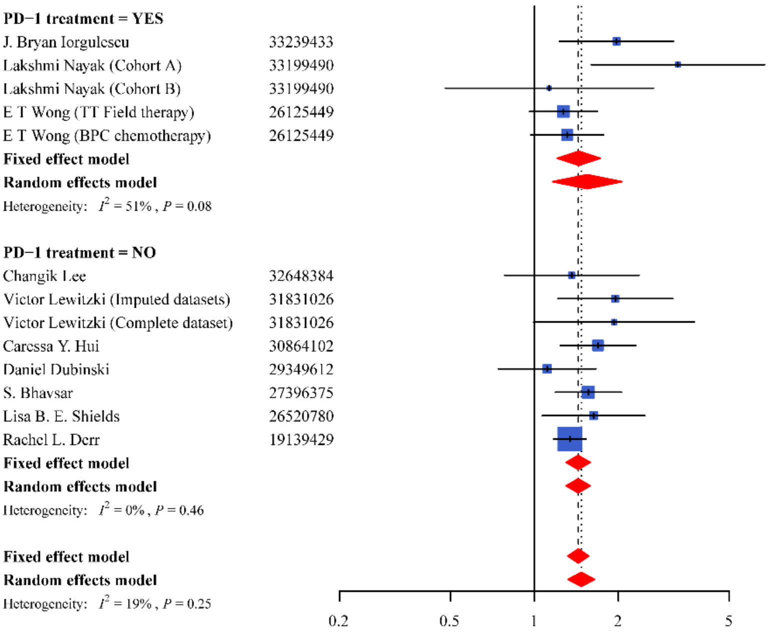

Study	PMID	HR	95%CI	Weight (fixed)	Weight (random)
**PD−1 treatment = YES**					
J. Bryan Iorgulescu	33239433	1.97	[1.23-3.16]	3.40%	4.79%
Lakshmi Nayak (Cohort A)	33199490	3.27	[1.60-6.69]	1.48%	2.22%
Lakshmi Nayak (Cohort B)	33199490	1.13	[0.48-2.68]	1.02%	1.56%
E T Wong (TT Field therapy)	26125449	1.27	[0.96-1.68]	9.56%	11.20%
E T Wong (BPC chemotherapy)	26125449	1.31	[0.97-1.77]	8.39%	10.15%
**Fixed effect model**		**1.44**	**[1.21-1.72]**	**23.85%**	—
**Random effects model**		**1.55**	**[1.16-2.06]**	—	**29.91%**
Heterogeneity: $I^2 = 51\%$, $P = 0.08$					
**PD−1 treatment = NO**					
Changik Lee	32648384	1.36	[0.78-2.37]	2.45%	3.56%
Victor Lewitzki (Imputed datasets)	31831026	1.95	[1.21-3.14]	3.35%	4.72%
Victor Lewitzki (Complete dataset)	31831026	1.93	[0.99-3.75]	1.71%	2.55%
Caressa Y. Hui	30864102	1.69	[1.24-2.31]	7.71%	9.51%
Daniel Dubinski	29349612	1.11	[0.74-1.66]	4.64%	6.27%
S. Bhavsar	27396375	1.56	[1.19-2.05]	10.02%	11.58%
Lisa B. E. Shields	26520780	1.63	[1.07-2.49]	4.22%	5.78%
Rachel L. Derr	19139429	1.34	[1.17-1.53]	42.05%	26.11%
**Fixed effect model**		**1.43**	**[1.30-1.58]**	**76.15%**	—
**Random effects model**		**1.43**	**[1.30-1.58]**	—	**70.09%**
Heterogeneity: $I^2 = 0\%$, $P = 0.46$					
**Fixed effect model**		**1.44**	**[1.32-1.57]**	**100.00%**	—
**Random effects model**		**1.47**	**[1.32-1.64]**	—	**100.00%**
Heterogeneity: $I^2 = 19\%$, $P = 0.25$					
